# Testicular Rupture Associated With an Unconventional Mechanism of Action

**DOI:** 10.7759/cureus.85769

**Published:** 2025-06-11

**Authors:** Matthew G Blalock, Kaitlyn M Flugga, Benjamin Reiswig, Bhavesh Joshi

**Affiliations:** 1 Department of Sports Medicine, Edward Via College of Osteopathic Medicine, Auburn, USA

**Keywords:** pole vault, testicular injury, testicular repair, testicular rupture, testicular ultrasound

## Abstract

Testicular and scrotal trauma is most commonly divided into two classifications based on the mechanism of action, blunt and penetrating, with penetrating trauma being reported more frequently. Blunt testicular injury is often due to motor vehicle accidents or contact sports where scrotal protection is not regularly worn, such as basketball or soccer.

Several hours after initially being struck in the left testicle by a fiberglass pole while vaulting, a 23-year-old man presented to the emergency department for testicular pain and swelling. Scrotal edema and ecchymosis were found on physical examination. Ultrasound findings showed a hydrocele with debris and structural changes within the testicle, suggestive of a testicular rupture. The patient was taken to the operating room for decompression of the hydrocele and further exploration. A testicular rupture was confirmed and repaired with partial resection of the seminiferous tubules. A follow-up appointment two weeks after surgery showed a well-healed incision without complication, and a semen analysis was completed at six weeks with normal results.

This case exhibits a unique mechanism of action for testicular rupture due to the lack of track-and-field-related trauma reported within the literature. Rapid diagnostic and surgical treatment were vital in this case to reduce the risk of fertility concerns and orchiectomy. Emergency medical providers must have a high index of suspicion for testicular rupture, given the severe complications if such a diagnosis is missed despite unusual mechanisms of injury.

## Introduction

Testicular trauma is a rare urologic emergency, with less than 1% of male trauma patients sustaining testicular or scrotal injury. Less than half of reported testicular injuries in the United States occur via blunt trauma, a majority of which involve high-velocity collisions, such as motor vehicle and biking accidents [1]. When testicular trauma occurs due to sports, the related injury almost exclusively occurs in a contact sport. The sports with the most frequent cases of testicular trauma from 2012 to 2021 included basketball (22.3%), football (21.8%), baseball (18.4%), and soccer (17.8%) [2]. Although the testicles are extracorporeal in nature, they are difficult to injure due to several biological mechanisms. The testes are situated in the scrotum but are not adhered to this outer layer, allowing for movement when pressure is applied via a blunt force. The tunica albuginea is a fibrous layer of collagen that surrounds each testis, acting as a protective layer [3]. Testicular rupture is defined as damage to the tunica albuginea with or without protrusion of seminiferous tubules through the defect [4]. The American Urological Association currently recommends surgical debridement of nonviable tissue in individuals with suspected testicular rupture and orchiectomy if the testicle is nonsalvageable [5]. One study found that delayed treatment of testicular rupture led to an orchiectomy rate of 45% compared to only 6% in those who underwent early exploration [6].

## Case presentation

**Figure 1:**
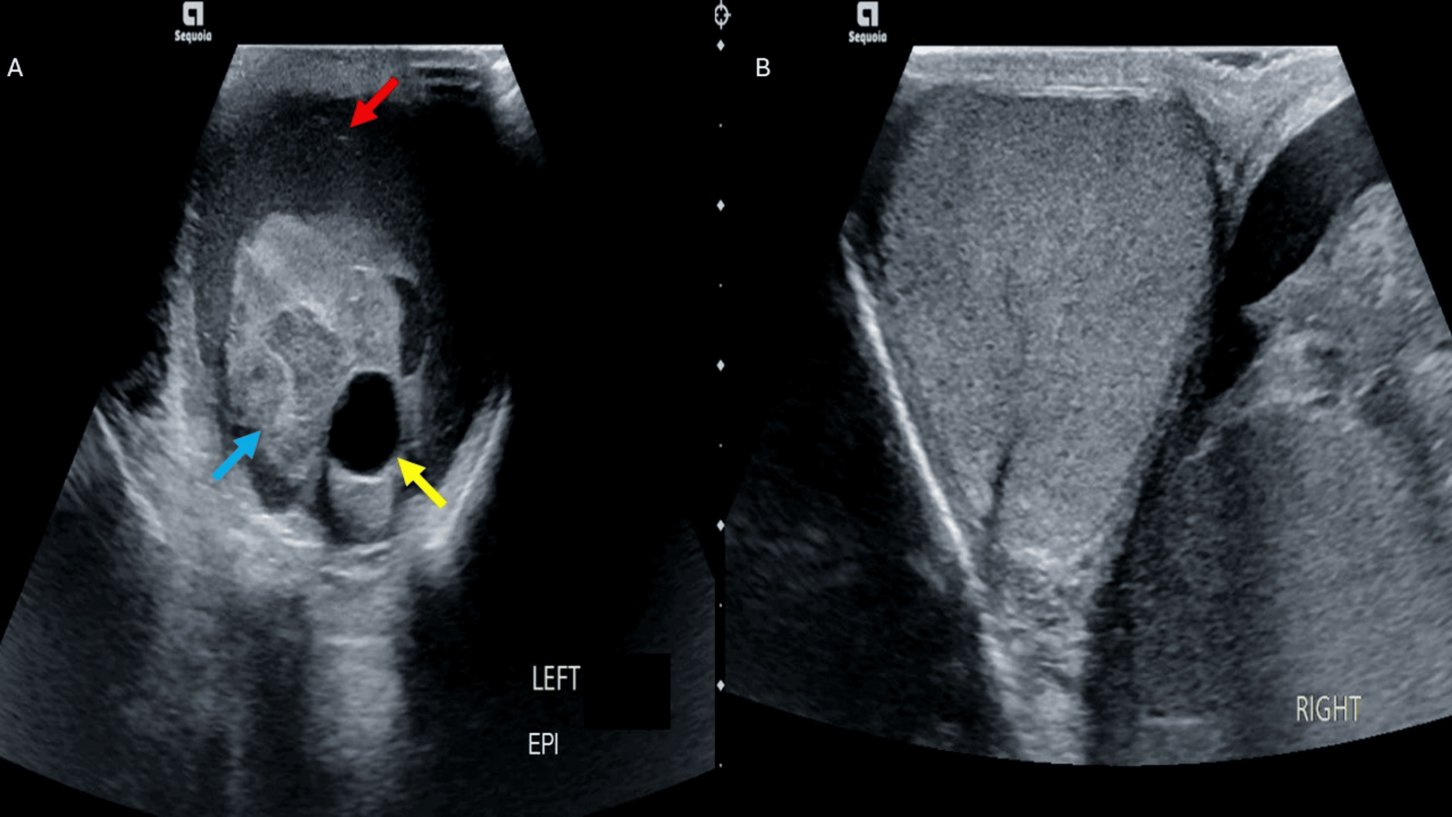
Testicular ultrasound images. (A) The left testicle has an intratesticular fluid collection (yellow arrow), hydrocele (red arrow), and heterogeneous parenchyma (blue arrow). (B) The right testicle demonstrates homogenous parenchyma without fluid pockets

A 23-year-old man presented to the emergency department with left-sided testicular pain and swelling. The patient was pole-vaulting when the fiberglass pole swung back, striking him in the left medial thigh and testicle. On physical examination, the left testicle had significant edema and ecchymosis. A testicular ultrasound was performed, which demonstrated a hydrocele and debris within the left testis with normal blood flow (Figure 1A). Ultrasonography of the right testis showed normal anatomy without signs of trauma (Figure 1B). The radiologist noted no definitive evidence of testicular fracture or torsion. Yet, debris is not routinely found within the testis and is suggestive of serious trauma as well as an intratesticular fluid collection with associated structural changes of the testicle.

Urology was consulted for assessment and potential decompression of the hydrocele noted on the ultrasound. After examining the patient, he was taken to the operating room for left scrotal exploration. There was approximately a two-hour time frame from presentation to surgical intervention. A roughly 5-cm incision was made at the median raphe of the scrotum. This was extended through the dartos fascia into the left hemiscrotum, where the tunica vaginalis was visualized. The tunica vaginalis was opened, which released a small to moderate amount of blood products. Upon inspection, a large transverse defect in the tunica albuginea across the anterior surface of the mid-testes was found. Exposed seminiferous tubules were protruding through the tunica albuginea, confirming the diagnosis of testicular rupture. The majority of the testicle was spared, with roughly 10%-20% loss of the total testicular volume in seminiferous tubules due to poor blood flow, followed by primary closure of the defect using 4-0 Prolene suture. The testicle was then placed back into the scrotum, with the dartos fascia and tunica vaginalis both being closed with a 2-0 chromic gut suture. The scrotal skin layer was then closed using 4-0 Monocryl in a running subcuticular fashion. Postoperative care consisted of a testicular support belt, a short course of trimethoprim-sulfamethoxazole, and follow-up in two weeks for evaluation. Two weeks postoperatively, the incision was well healed without erythema or evidence of hydrocele. Six weeks after surgery, a semen analysis was completed for the evaluation of fertility. Although there were no previous seminal samples for comparison, no obvious fertility concerns were identified due to this patient’s case of testicular rupture.

## Discussion

Unlike most testicular trauma, which stems from high-impact forces like motor vehicle accidents or gunshot wounds, this case demonstrates the need for serious investigation into all groin injuries due to its unusual nature. Even among injuries sustained during sports, testicular trauma remains very rare, with only 0.24% of emergency room visits being due to genital injury in athletes [2]. This low rate of occurrence during physical activity is most likely secondary to the implementation of protective equipment like athletic cups and groin guards.

Physical examination of those with potential testicular rupture may be difficult due to pain, but it is necessary in combination with imaging to successfully diagnose these injuries. Similar to the physical examination seen in our patient, the scrotum is often swollen, with ecchymosis and sensitivity to touch [7,8]. The cremasteric reflex should remain intact for those with testicular rupture, as there is no damage to the spermatic cord or genitofemoral nerve that corresponds with this reflex.

Rapid assessment and surgical intervention were key in this case to reduce patient complications such as atrophy of the testicle, subfertility and infertility, and orchiectomy due to ischemia and necrosis. Ultrasound remains the mainstay for diagnosing testicular injury due to its accessibility; however, it can be limited by the technician's expertise in performing the imaging [9]. A high-frequency transducer is recommended to evaluate the tunica albuginea successfully [10]. Testicular rupture on ultrasonography demonstrates heterogeneity of the parenchyma and visualization of the ruptured tunica albuginea, and in 80% of cases, a hematocele or hydrocele is present [11,12]. While more difficult to obtain, magnetic resonance imaging (MRI) has made its way to the forefront of testicular imaging as the most accurate methodology. Current guidelines only recommend MRI for acute testicular trauma if sonographic results are inconclusive with a high level of suspicion for rupture or torsion [13]. Due to both the high cost and difficulty in obtaining an MRI in a timely manner, ultrasound remains the mainstay diagnostic imaging for testicular and genital trauma.

**Figure 2:**
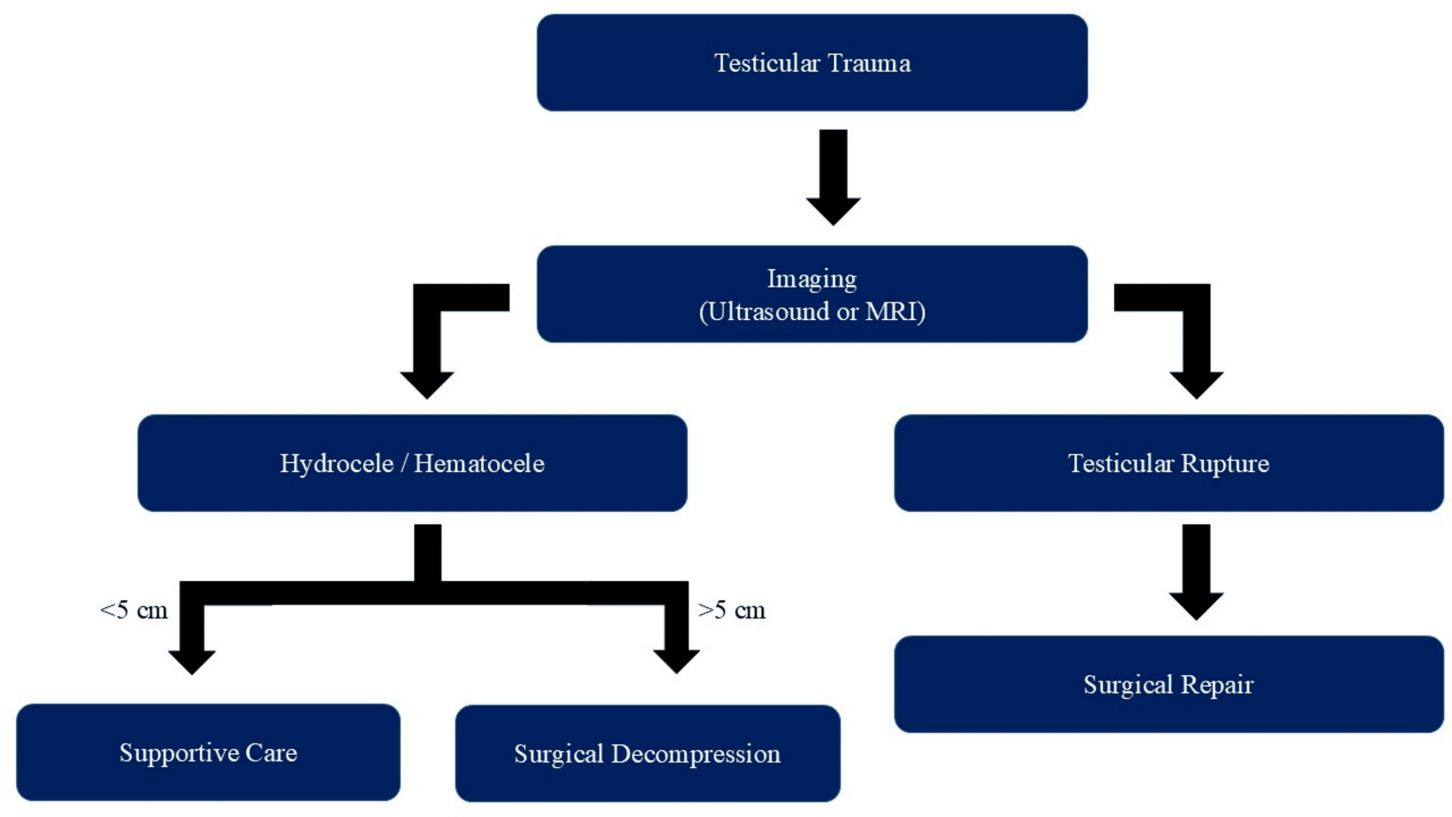
Flowchart of surgical candidacy for testicular injury On initial presentation, testicular imaging should be performed. If there are definitive signs of testicular rupture, the patient should be taken to the operating room for repair. If a hematocele is present without signs of growth and is measured to be less than 5 cm, then conservative management is an option. If the hematocele does not meet the previously stated criteria, surgical decompression of the testis should be performed

The American Urological Association’s guidelines state scrotal exploration should be performed in those with suspected testicular rupture [5]. Surgical repair of testicular rupture within the first 72 hours has shown a testicular salvage rate of 90%-100% compared to 45%-55% with delayed surgery, with one study showing an orchiectomy rate of 45% in those who delayed treatment [12,14]. Testicular rupture can be treated surgically via orchiectomy if the testicle is not grossly viable or by dissection of necrotic or ischemic tissue with closure of the tunica albuginea. If the tunica albuginea cannot be closed, graft tissue from the tunica vaginalis may be used to cover the defect completely [8]. A synthetic graph has also been used, although increased rates of infection have been reported. For hematoceles without definitive signs of rupture, conservative management can be performed if the hematocele is less than 5 cm and nonexpanding or if the injured testicle is less than three times the size of the contralateral testis [3]. A visual explanation has been provided below for the clinical decision-making criteria for surgical intervention of a testicular rupture (Figure 2).

After surgical intervention, patients should receive a course of antibiotics, a scrotal support system, and pain management [4]. Following up with a urologist after surgical repair for testicular rupture is also recommended for an ultrasound to assess blood flow to the testis, as well as to confirm no further hydrocele has developed. If the patient is concerned about future reproductive capabilities, semen analysis can be considered.

## Conclusions

Testicular injury secondary to pole-vaulting is an extremely rare phenomenon not frequently reported due to a lack of contact within the sport that would put the testicle at risk for blunt trauma. The time from initial injury to assessment is vital in these cases, with poor outcomes reported when a delay in definitive treatment was reported. Sonography is the first-line choice of imaging with scrotal injury as it is the fastest and most easily accessible option in an emergency setting. Whenever possible, testicular sparing treatment should be attempted first before performing an orchiectomy in order to reduce the risk of subfertility. If there are fertility concerns after surgical intervention, semen analysis may be performed four to six weeks postoperatively to allow appropriate time for healing.
